# Depot-Specific Changes in Fat Metabolism with Aging in a Type 2 Diabetic Animal Model

**DOI:** 10.1371/journal.pone.0148141

**Published:** 2016-02-19

**Authors:** Se Eun Park, Cheol-Young Park, Jung Mook Choi, Eugene Chang, Eun-Jung Rhee, Won-Young Lee, Ki Won Oh, Sung Woo Park, Eun Seok Kang, Hyun Chul Lee, Bong Soo Cha

**Affiliations:** 1 Division of Endocrinology and Metabolism, Department of Internal Medicine, Kangbuk Samsung Hospital, Sungkyunkwan University School of Medicine, Seoul, Korea; 2 Diabetes Research Institute, Kangbuk Samsung Hospital, Sungkyunkwan University School of Medicine, Seoul, Korea; 3 Division of Endocrinology and Metabolism, Department of Internal Medicine, Yonsei University, College of Medicine, Seoul, Korea; University of Birmingham, UNITED KINGDOM

## Abstract

Visceral fat accretion is a hallmark of aging and is associated with aging-induced metabolic dysfunction. PPARγ agonist was reported to improve insulin sensitivity by redistributing fat from visceral fat to subcutaneous fat. The purpose of this study was to investigate the underlying mechanisms by which aging affects adipose tissue remodeling in a type 2 diabetic animal model and through which PPARγ activation modulates aging-related fat tissue distribution. At the ages of 21, 31 and 43 weeks, OLETF rats as an animal model of type 2 diabetes were evaluated for aging-related effects on adipose tissue metabolism in subcutaneous and visceral fat depots. During aging, the ratio of visceral fat weight to subcutaneous fat weight (V/S ratio) increased. Aging significantly increased the mRNA expression of genes involved in lipogenesis such as lipoprotein lipase, fatty acid binding protein aP2, lipin 1, and diacylglycerol acyltransferase 1, which were more prominent in visceral fat than subcutaneous fat. The mRNA expression of adipose triglyceride lipase, which is involved in basal lipolysis and fatty acid recycling, was also increased, more in visceral fat compared to subcutaneous fat during aging. The mRNA levels of the genes associated with lipid oxidation were increased, whereas the mRNA levels of genes associated with energy expenditure showed no significant change during aging. PPARγ agonist treatment in OLETF rats resulted in fat redistribution with a decreasing V/S ratio and improved glucose intolerance. The genes involved in lipogenesis decreased in visceral fat of the PPARγ agonist-treated rats. During aging, fat distribution was changed by stimulating lipid uptake and esterification in visceral fat rather than subcutaneous fat, and by altering the lipid oxidation.

## Introduction

Fat tissue is at the nexus of mechanisms and pathways involved in longevity, genesis of age-related disease, inflammation and metabolic dysfunction. Fat mass and fat tissue distribution change dramatically throughout life [1,2]. In old age, while the total amount of fat tissue tends to decline or remain stable, fat distribution changes dramatically. By advanced old age, fat is redistributed from subcutaneous to intra-abdominal visceral deposits and to ectopic sites, including muscle, liver and bone marrow [3–5]. These changes are associated with diabetes, hypertension, cancer, cognitive dysfunction and atherosclerosis [1,2]. Several studies also suggest that insulin resistance and altered glucose homeostasis are more closely related to regional adipose tissue distribution than total fat mass [6,7].

Visceral fat releases more nonesterified fatty acids into circulation than subcutaneous fat [8], which is liable to expose the liver to high amounts of nonesterified fatty acids and lead to increased hepatic glucose production and VLDL secretion [9]. High plasma nonesterified fatty acids lead to lipid accumulation in nonadipose tissue and interferes with insulin signaling [10,11]. In addition, enlarged visceral fat deposits secrete a wide range of proinflammatory cytokines that reduce insulin signaling and promote endothelial dysfunction [12]. However, there are few data about the changes of visceral fat and related adipose tissue metabolism with aging.

Peroxisome proliferator–activated receptor (PPARγ) is a ligand-activated nuclear receptor that is highly expressed in mammalian white adipose tissue (WAT), where it regulates the expression of a number of genes involved in lipid and glucose metabolism. PPARγ agonists of the thiazolidinedione (TZD) class are currently used for treatment of insulin resistance and type 2 diabetes. The mechanisms involved in the insulin-sensitizing effect of PPARγ agonists are not completely understood but appear to involve changes in regional adiposity by favoring lipid accumulation in subcutaneous fat, while reducing or maintaining visceral fat mass [13,14]. More specifically, PPARγ activation in both humans and rodents is associated with an enhanced ability for subcutaneous fat to take-up and store fatty acids, especially those derived from lipoprotein-bound triacylglycerol through lipoprotein lipase [15]. Directing fat away from visceral fat to subcutaneous fat deposits may constitute one mechanism whereby PPARγ agonism prevents the deleterious effects of visceral fat accumulation on the development of metabolic syndrome and the progression to cardiovascular disease. These depot-specific effects might be helpful for preventing the changes of adipose tissue metabolism with aging. However, the PPARγ agonism on the aging-related changes of fat metabolism has not been addressed in detail.

Fat storage represents the balance between accretion (uptake, synthesis and esterification) and depletion (release of lipolytic products, oxidation and energy-consuming cycling). The goal of this study was to establish which of these pathways is associated with age-related depot-specific fat accretion in a rodent model of obesity and type 2 diabetes. To this end, determinants of adipose lipid metabolism were assessed in subcutaneous and visceral fat, including triglyceride-derived fatty acid uptake and retention, lipolysis, fatty acid reesterification, lipid oxidation and energy expenditure. The levels of expression of major genes associated with these pathways were quantified, and the effect of PPARγ activation in vivo on fat tissue distribution was compared with aging-related changes.

## Methods and Procedures

### 1. Animals

Laboratory animals for all experiments were cared for in accordance with the National Institute of Health’s guidelines. The animals were maintained according to the ethical guidelines of Yonsei University, and the experimental protocol was approved by the Committee on Animal Investigations of Yonsei University. Male Otsuka Long-Evans Tokushima Fatty (OLETF) rats (70–80 g initial weight) and their nondiabetic counterparts, male Long-Evans Tokushima Otsuka (LETO) rats, were supplied by the Tokushima Research Institute, Otsuka Pharmaceutical (Tokushima, Japan). We used the OLETF rats, as a model for aging because they have age associated changes to body fat contents and metabolic derangement (16). The LETO rats showed no significant changes to fat distribution and glucose tolerance in respect to aging (S1 Fig). They were maintained in the Animal Care Center at the University of Yonsei Medical School, Seoul, South Korea, under controlled temperature (23°C ± 2°C) and humidity (55% ± 5%) with a 12-hour light/dark cycle. The animals were provided standard rat chow and tap water ad libitum.

### 2. Experimental Protocol

The standard rat diet had an energy content of approximately 15.1 kJ/g (3.6 kcal/g) and contained 21% protein, 12.5% fat and 66.5% carbohydrates. The OLEFT rats were randomly divided into four groups: (1) OLETF rats treated with the PPARγ agonist rosiglitazone (4mg/kg per day) for six weeks at 21 weeks of age (n = 8), (2) OLEFT rats without rosiglitazone treatment at 21 weeks of age (n = 8), (3) OLETF rats at 31 weeks of age (n = 8), (4) OLETF rats at 43 weeks of age (n = 5). The age of the week of the OLEFT rat was selected according to diabetes status [16]. Each group consisted of three to eight animals.

At 15 weeks of age, six OLETF rats were randomly selected for treatment with rosiglitazone. The allocated diet and drug treatment was maintained for the following six weeks. Rosiglitazone, or distilled water as a placebo, was given by oral gavage. Two rats were assigned to each cage. All animals were allowed free access to food and water throughout the study and were weighed weekly; food intake was determined every two days. Mean food intake was estimated as an average for the animals in each cage. After six weeks of treatment, an oral glucose tolerance test (OGTT) was performed after an overnight fast. One day after the OGTT, the OLETF rats were anesthetized with ether and sacrificed. The LETO rats at 21 weeks and the untreated OLEFT rats at ages 21, 31 and 43 weeks were also sacrificed after OGTT.

Epididymal fat as a representative of visceral fat [17] and abdominal subcutaneous fat were surgically removed after mid-abdominal incision. Each dissected fat mass was immediately weighed and stored at −80°C until the assays were performed. A portion of the pancreas was fixed in 10% neutral buffered formalin.

### 3. Blood and tissue collection

Blood samples were obtained from the heart at the time of sacrifice and were immediately centrifuged at 5000x g for 5 min. Total cholesterol concentrations were determined using an ADVIA 1650 (Bayer, West Haven, CT, USA). Plasma triglyceride and glucose concentrations were measured by a commercial kit (SekisuiI Chemical Company, Osaka, Japan). Free fatty acid (FFA) levels were measured by enzymatic assay kit from SCIDIA NEFAZYME (Shinyang Diagnostics, Seoul, Korea). Commercially available ELISA kits were used to measure insulin (Millipore, Billerica, MA, USA).

### 4. OGTT

Rats were orally given glucose (2g/kg), and blood samples were collected from the tail at 0, 60 and 120 minutes after glucose load. Glucose levels were measured with a glucose analyzer (SureStep, Lifescan, Milpitas, CA).

### 5. Real-time reverse transcriptase polymerase chain reaction (RT-PCR)

Total RNA was isolated from cells and tissues with the use of a PureLink RNA Mini Kit (Invitrogen). Reverse transcription was performed using a High-Capacity RNA-to-cDNA kit (Applied Biosystems, Foster City, CA, USA) per the manufacturer's instructions. mRNA expression was quantified by real-time PCR (LightCycler 480 system; Roche, Indianapolis, IN, USA). Synthesized cDNA was mixed with LightCycler 480 Probes Master Mix (Roche) and with a gene-specific primer and probe mixture (Universal ProbeLibrary system and UPL; Roche; Table 1). Individual reactions for target and glyceraldehyde-3-phosphate dehydrogenase (*Gapdh*) were carried out separately with negative controls lacking cDNA. Reaction conditions were as follows: 95°C for 10 min, followed by 40 cycles of denaturation (95°C for 10 s) and annealing/extension (60°C for 20 s). The cycle number for the threshold of detection was determined by LightCycler 480 software (Roche). mRNA expression of each target was normalized to that of the *Gapdh* gene and expressed as a fold-change relative to the controls (primers listed in Table 1).

**Table 1:** Sequences of primers and PCR reaction parameters used in real-time RT-PCR.

Target gene	Forward	Reverse	AT (°C)
**LPL**	AACCTTTGTGGTGATCCATGGA	CGAAATCCGCATCATCAGGA	60
**aP2**	ATGTGTGATGCCTTTGTGGG	CCCAGTTTGAAGGAAATCTC	55
**Lipin1**	TCACTACCCAGTACCAGGGC	TGAGTCCAATCCTTTCCCAG	55
**DGAT1**	TATTACTTCATCTTTGCTCC	AAAGTAGGTGACAGACTCAG	45
**PEPCK**	TGGGTGATGACATTGCCTGG	ACCTTGCCCTTATGCTCTGCAG	60
**ATGL**	CATTTTAGCTCCAAGGATGA	TGGTTCAGTAGGCCATTCCT	55
**PDK2**	GGGGTGTCCCCTTGAGGAAGAT	TTCTTGGGCTCTGTGCTGGG	55
**mCPT1**	CGGAAGCACACCAGGCAGTA	GCAGCTTCAGGGTTTGTCGGAATA	60
**LCAD**	AGCTCCCACAGGAAAGGCTT	CTCGAGCATCCACGTAGGCT	55
**GAPDH**	TGAACGGGAAGCTCACTGG	TCCACCACCCTGTTGCTGTA	60

### 6. Histologic analysis and adipocyte cell size distribution

Adipose tissues were fixed overnight in 10% (vol./vol.) zinc formalin, dehydrated in a graded series of alcohol washes, cleared in toluene and embedded in paraffin. Using a microtome, 5 μm sections were generated, collected on slides, and then stained with hematoxylin and eosin. Samples of subcutaneous and epididymal adipose tissues were fixed in 10% formalin and embedded in paraffin. Multiple sections (separated by 100 μm each) were obtained from each sample and stained with hematoxylin and eosin. Digital images of each section were acquired using a BX51TRF microscope (Olympus, Japan), and cell areas were traced manually for at least 100 cells per field by an investigator blinded to the sample identity, using the ImageJ software program (available at http://rsb.info.nih.gov/ij/). Two fields from each section of adipose tissue depot were analyzed to derive the mean cell area per animal (*n* = 3 animals per group).

### 7. Statistical analyses

All statistical analyses were performed using PASW Statistics 18 (SPSS Inc., Chicago, IL, USA). Data are expressed as mean±SEM. We used 2-way ANOVA with turkey posthoc analysis to assess the effects of aging (at 21, 31, and 43 weeks old) and/or fat depots (subcutaneous fat vs. visceral fat) on the adipose tissue metabolisms in the OLETF rats. We performed 1-way ANOVA with posthoc analysis to compare the effects of rosiglitazone on the metabolic genes in each fat depot among the different groups at 21 weeks of age. Statistical significance was defined as *P*<0.05.

## Results

### 1. Changes in glucose metabolism and fat distribution with age

At 21 weeks of age, the OLETF rats had a significantly higher body weight compared with the LETO rats. Serum levels of glucose, insulin, triglyceride and FFA increased in the OLETF rats compared to the LETO rats. From 21 to 43 weeks of age, body weight significantly increased with age in the OLEFT rats. Aging also contributed to increased fasting glucose, fasting insulin, triglyceride and FFA levels in the OLETF rats (Table 2).

**Table 2:** Comparison of metabolic parameters measured in rats with aging or PPARγ agonist treatment.

	LETO rats	OLETF rats			
	21 weeks (n = 4)	21 weeks (n = 8)	31 weeks (n = 8)	43 weeks (n = 5)	21 weeks PPARγ agonist (n = 8)
**Body weight (g)**	463.3±14.7 *	586.6±11.0	614.2±19.2	620.0±40.0	608.9±38.3
**Subcutaneous fat (% body weight)**	0.7±0.1 *	2.6±0.7	4.8±0.3*	4.7±0.8*	3.2±0.3
**Visceral fat (% body weight)**	0.8±0.1 *	3.3±0.5	11.0±1.2*	12.9 ±1.2*	2.4±0.5*
**V/S ratio**	1.1±0.1 *	1.3±0.1	2.3±0.1 *	2.8±0.4*	0.8±0.1 *
**Glucose (mmol/l)**	7.1±0.7*	11.6±0.4	12.1±0.3	16.9±0.1*	9.8±0.5 *
**Insulin (ng/ml)**	155.8±68.3*	511.8±75.7	566.0±356.6	708.6±383.4*	409.9±20.1*
**Free fatty acid (mmol/l)**	524.0±90.6 *	944.1±62.5	958.7±30.2	1053.3±51.5*	517.8±52.8 *
**Triglyceride (mmol/l)**	0.2±0.0*	1.6±0.3	2.3±0.6	2.0±0.2	0.3±0.0*

Data are summarized in mean ± SEM.

*P<0.05, compared with OLETF rats at 21 weeks.

**Fig 1:**
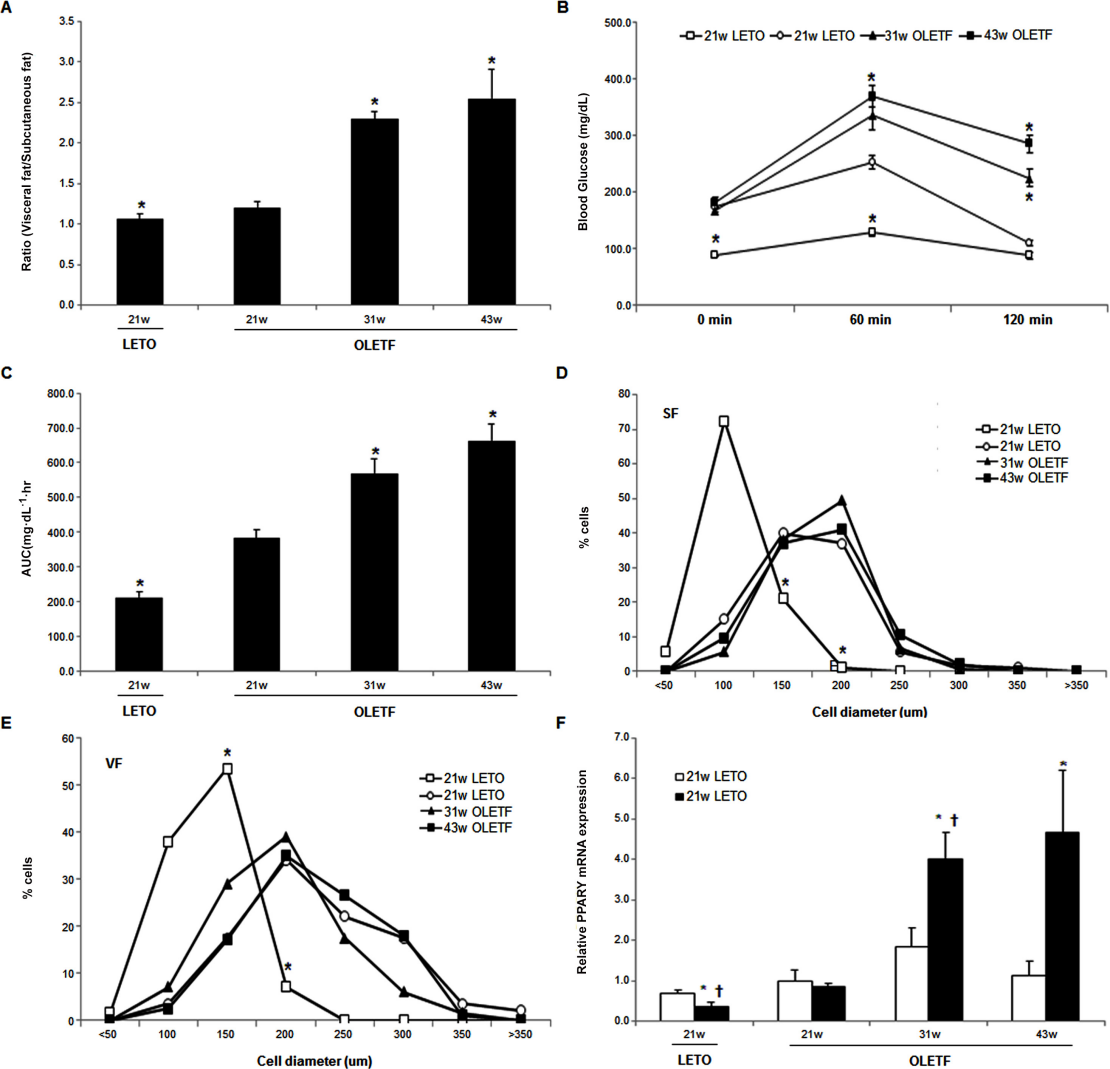
**The effect of aging on the weight of subcutaneous fat/visceral fat ratio (A), OGTT (B), AUC during OGTT (C), adipocyte size distribution of subcutaneous fat (D) and visceral fat (E), and PPARγ2 mRNA levels (F).** (*P <0.05 vs. the same deposit in the untreated OLETF rats, ^†^*P*<0.05 vs. subcutaneous fat in the same group.)

The ratio of visceral fat weight to subcutaneous fat weight (V/S ratio) was higher in the OLETF rats than in the LETO rats (Fig 1A, Table 2). In terms of area under the curve (AUC) during the OGTT, the OLETF rats showed decreased glucose utilization compared to the LETO rats (Fig 1A–1C). As the OLETF rats aged, V/S ratio increased as did the AUC for the OGTT (Fig 1A–1C, Table 2). Compared with OLETF rat at the same age, adipocyte size were smaller in LETO rats in both subcutaneous fat and visceral fat. Aging also brought about the shift in adipocyte size distribution toward larger cell diameters in both types of fat depots (Fig 1D and 1E). PPARγ2 expression levels in visceral fat were higher in the OLEFT rats than in the LETO rats and increased with age (Fig 1F).

### 2. Determinants of adipose fatty acid uptake, esterification, and triacylglycerol synthesis

**Fig 2:**
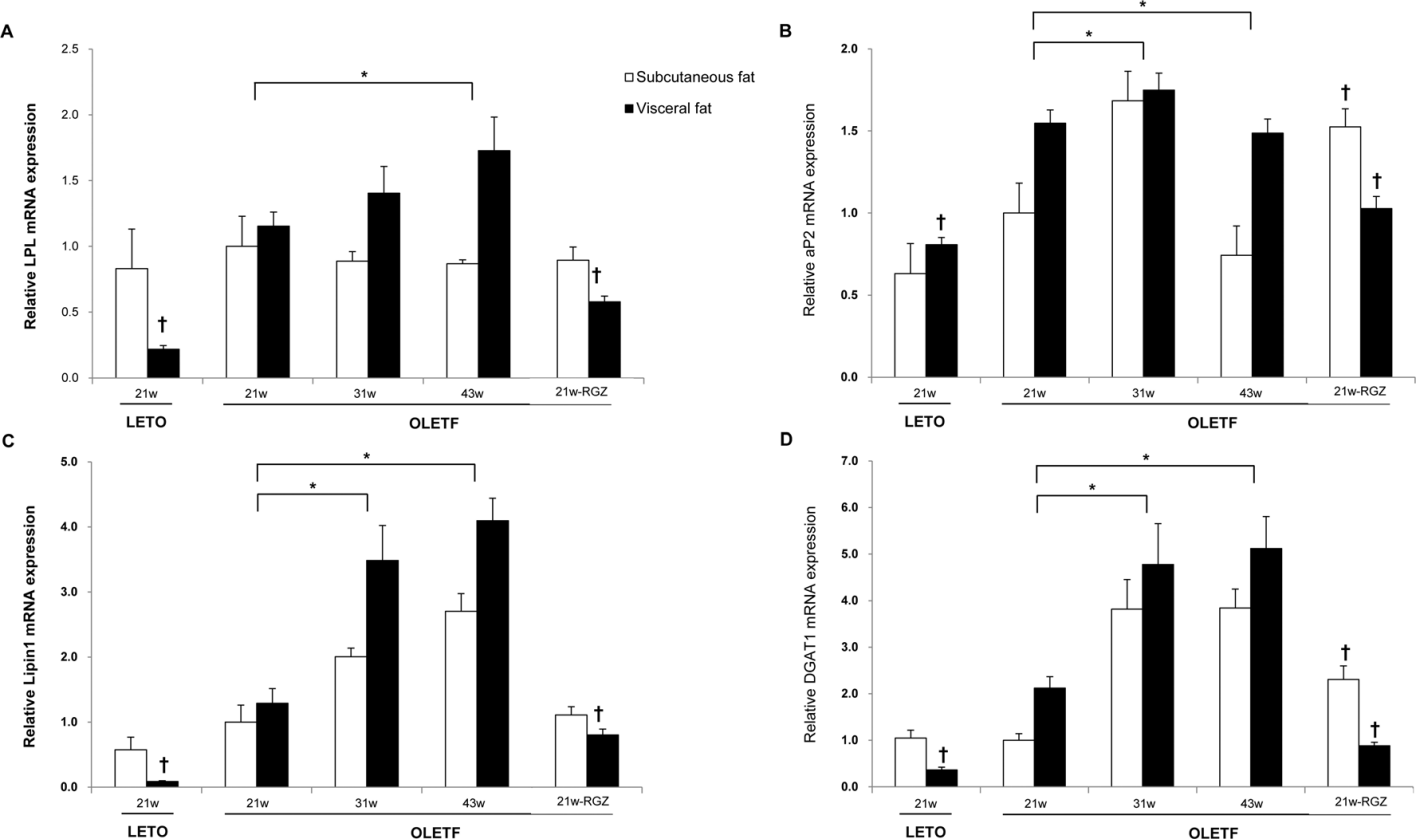
The effects of aging on the genes involved in adipose fatty acid uptake, esterification and triacylglycerol synthesis. The effects of aging on the gene expression were analyzed by 2-way ANOVA (*P <0.05 vs. OLETF rats at 21 weeks of age). The effects of rosiglitazone on the metabolic genes in each fat depot were evaluated among the different groups at 21 weeks (†*P* <0.05 vs. the same deposit in OLETF rats at 21 weeks; RGZ, rosiglitazone treated).

The expression of genes involved in fatty acid utilization and triacylglycerol synthesis were examined to gain insight into the mechanisms that influence the effects of aging on adipose tissue remodeling. Compared with the visceral fat of LETO rats at age 21 weeks, gene expression of the triglyceride-hydrolyzing enzyme lipoprotein lipase (LPL) significantly increased in OLETF rats of the same age (Fig 2A). The fatty acid binding protein aP2 is a major PPARγ target during adipogenesis, long-chain fatty acid uptake, and retention [18], and mRNA levels of this protein also increased in the visceral fat of OLETF rats compared to LETO rats (Fig 2B).

Lipin 1, which has been identified as phosphatidate phosphatase-1 (PAP1), catalyzes the conversion of phosphatidate to diacylglycerol during triacylglycerol and phospholipid biosynthesis [19]. Diacylglycerol acyltransferase-1 (DGAT-1) catalyzes the terminal, rate-limiting step in triglyceride synthesis, and its overexpression favors fat gain [20]. Both lipin-1 and DGAT-1 mRNA were more highly expressed in the visceral fat of OLETF rats than LETO rats at 21 weeks of age (Fig 2C and 2D). On the other hand, there were no significant differences in mRNA expression of LPL, aP2, lipin 1 or DGAT-1 in subcutaneous fat when comparing LETO and OLETF rats (Fig 2A–2D).

To evaluate the significant effects either of aging and/or fat depots on the mRNA expression, the data were further assessed by 2-way ANOVA. In OLETF rats, both aging and fat distribution have significant effects on the mRNA levels of LPL, fatty acid binding protein aP2, lipin1, and DGAT1 (all *P*<0.05).

Compared with the 21-week-old OLETF rats, the mRNA levels of LPL significantly increased at 43 weeks of age (Fig 2A). At 31 and 43 weeks of age, OLETF rats had increased mRNA expression of aP2, lipin1 and DGAT1 than those at 21 weeks of age (Fig 2B–2D). The fat distribution also had statistically significant effects on the expression of these genes. The mRNA levels of the LPL and aP2 linearly increased in visceral fat deposits with aging, but not in subcutaneous fat deposits (Fig 2A and 2B). The mRNA expressions of lipin1 and DGAT1 in both subcutaneous and visceral fat deposits were increased, and the changes were more dramatic in visceral fat. The mRNA expressions of lipin1 and DGAT1 in subcutaneous and visceral fat showed a 2.7- vs. 3.2-fold and 2.2- vs. 2.7-fold difference, respectively (Fig 2C and 2D).

### 3. Determinants of glycerol and fatty acid cycling

**Fig 3:**
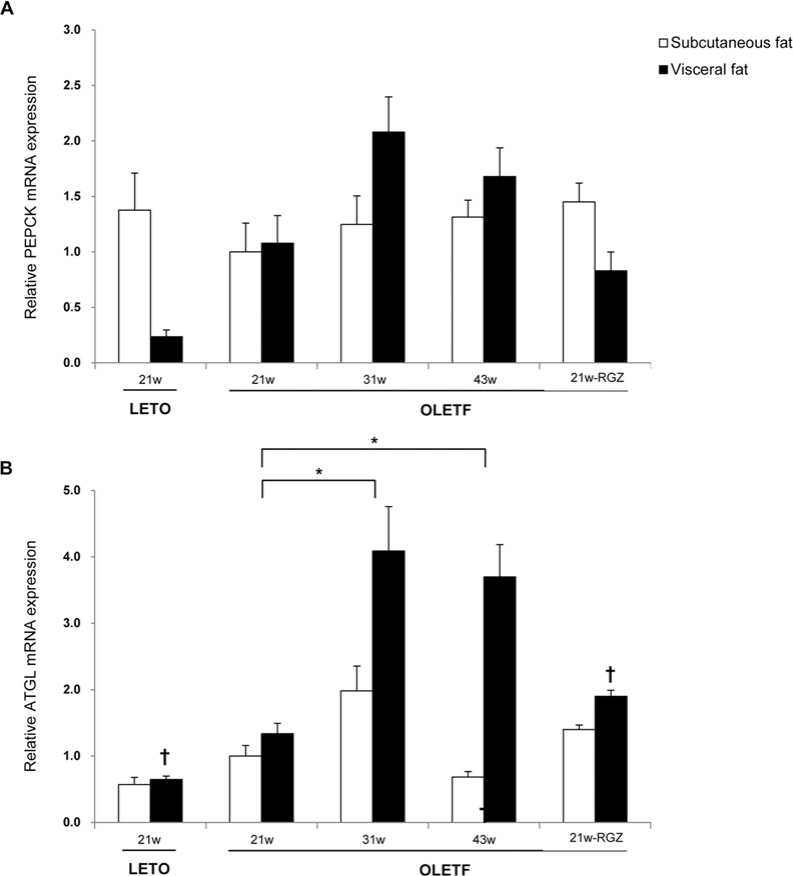
The effects of aging on the genes involved in glycerol and fatty acid recycling. The effects of aging on the gene expression were analyzed by 2-way ANOVA (*P <0.05 vs. OLETF rats at 21 weeks of age). The effects of rosiglitazone on the metabolic genes in each fat depot were evaluated among the different groups at 21 weeks (†*P* <0.05 vs. the same deposit in OLETF rats at 21 weeks; RGZ, rosiglitazone treated).

There was no difference between the mRNA levels of phosphoenolpyruvate carboxykinase (PEPCK), which is related to glyceroneogenesis [21], in subcutaneous and visceral fat deposits of the OLETF and LETO rats. Also, aging and fat deposits had no significant effects on the mRNA expression of PEPCK in the OLETF rats (Fig 3A). Adipose triglyceride lipase (ATGL) mRNA expression, considered an important determinant of basal lipolysis [22], was highly expressed in the visceral fat of the OLETF rats at 21 weeks compared to the LETO rats. The mRNA levels of ATGL significantly increased in 31 and 43 weeks-old OLETF rats compared to the rats at 21 weeks of age. The fat redistribution also had significant effects on the changes of ATGL mRNA levels during aging. The mRNA levels of ATGL increased more in visceral fat than in subcutaneous fat (Fig 3).

### 4. Determinants of fatty acid oxidation and energy expenditure

**Fig 4:**
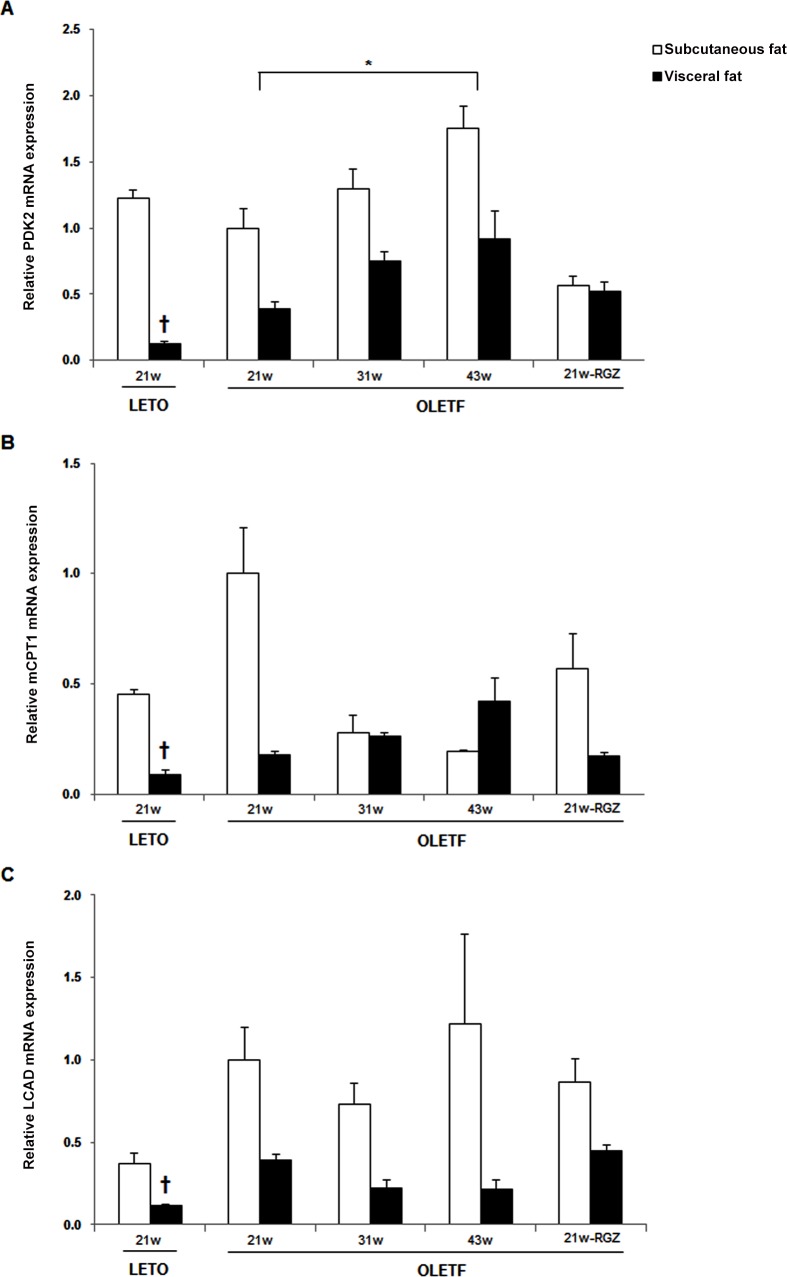
The effects of aging on the genes involved in fatty acid oxidation and energy expenditure. The effects of aging on the gene expression were analyzed by 2-way ANOVA (*P <0.05 vs. OLETF rats at 21 weeks of age). The effects of rosiglitazone on the metabolic genes in each fat depot were evaluated among the different groups at 21 weeks (†*P* <0.05 vs. the same deposit in OLETF rats at 21 weeks; RGZ, rosiglitazone treated).

Pyruvate dehydrogenase kinase-2 (PDK2) phosphorylates and inactivates the pyruvate dehydrogenase complex and thereby facilitates fatty acid oxidation. PDK-2 mRNA expression in visceral fat was significantly higher in the OLETF rats than in the LETO rats, though there were no significant differences in subcutaneous fat between LETO and OLETF rats at 21 weeks of age (Fig 4A). Muscle-type carnitine palmitoyltransferase 1 (mCPT-1), the limiting enzyme in fatty acid transport to mitochondria, significantly increased in visceral fat of OLETO rats than LETO rats at 21 weeks of age (Fig 4B). Expression of long-chain acyl-CoA dehydrogenase (LCAD), which plays an important role in β-oxidation, also increased in the visceral fat of OLETF rats compared to LETO rats of the same age (Fig 4C).

Among the genes associated with fatty acid oxidation and energy expenditure, aging had effects on PDK-2 but not on mCPT1 and LCAD mRNA levels. Compared to rats at 21 weeks of age, the mRNA levels of PDK-2 at 43 weeks were increased. Additionally, the PDK-2, mCPT1, and LCAD mRNA levels were all significantly different between the fat depots (Fig 4A–4C).

### 5. PPARγ agonist might modify age-related fat distribution

Six weeks of rosiglitazone treatment in OLETF rats tended to increase body weight and rate of body weight gain. Compared with untreated OLETF rats at the same age, rosiglitazone treatment had no influence on body weight and food intake. At 21 weeks of age, the V/S ratio was significantly lower in the rosiglitazone-treated OLETF rats than in the untreated OLETF rats. In the rosiglitazone-treated OLETF rats, serum levels of fasting glucose, fasting insulin, triglycerides and FFA were significantly lower than in the untreated OLETF rats (Table 2). As expected, the rosiglitazone-treated OLETF rats showed improved glucose utilization compared to untreated OLEFT rats using the AUC during the OGTT. Rosiglitazone administration induced smaller adipocyte size in both subcutaneous and visceral fat deposits compared with untreated rats (S2 Fig).

After confirming major fat deposit-specific differences in fatty acid handling and triacylglycerol synthesis with aging, this study then investigated whether this specificity was modified by PPARγ action. Compared with untreated rats at 21 weeks of age, rats treated with rosiglitazone exhibited significantly decreased mRNA expression of key enzymes in visceral fat, including LDL, aP2, lipin1 and DGAT1 (Fig 2). In contrast, levels of mRNA expression in these genes remained the same or slightly increased in the subcutaneous fat of rosiglitazone-treated OLETF rats (Fig 2). Rosiglitaonze had no significant effects on the PEPCK expression in both fat depots (Fig 3A). The mRNA expressions of ATGL increased with rosiglitazone treatment in visceral fat (Fig 3B). In addition, rosiglitazone did not affect the expression of genes involved in fatty acid oxidation or energy expenditure (PDK2, mCPT-1 and LCAD) in any of the fat deposits (Fig 4).

## Discussion

This study demonstrated the effect of aging on the deposit-specific regulation of lipid storage and energy expenditure genes in a type 2 diabetic animal model. Aging led to changes in fat distribution by increasing lipid uptake and esterification and altering energy expenditure in visceral fat compared to subcutaneous fat. Conversely, the PPARγ agonist rosiglitazone may affect adipose tissue distribution to subcutaneous deposits by changing several pathways of adipose lipid metabolism. These results suggests that the deleterious effects of fat distribution with aging might be partially modulated by PPARγ agonists such as rosiglitazone.

Aging increased cell size and led to a substantial redistribution of fat tissue in this type 2 diabetic animal model. Serum levels of glucose, insulin and FFA also increased with aging, which might result from aging-increased visceral fat pads and the associated adipocyte metabolism pathways of lipogenesis and lipolysis [23]. In this study, there were similar trends in genes involved in lipogenesis. The expression of PPARγ and its target genes were increased predominantly in visceral fat with aging. The mRNA levels of LPL increased in visceral fat due to the effects of aging. Genes such as aP2, lipin1 and DGAT1 also increased to a greater extent in visceral fat deposits than in subcutaneous fat deposits. However, there were no significant changes in the expression levels of PEPCK, which is associated with fatty acid recycling [21], during aging. Higher lipolysis is counteracted by increased fatty acid reesterification into triglycerides, as well as by increased fatty acid reuptake by adipocytes [24,25]. Together, these results show that ATGL mRNA expression was significantly higher in visceral fat than in subcutaneous fat, and aging increases this gene expression. Elevated FFAs were recently shown to be associated with an increase in PEPCK–mediated glyceroneogenesis in WAT [21]. These data suggest that aging is related to a cycle between lipogenesis and lipolysis in adipose tissue metabolism in visceral fat. Aging might remodel the adipose tissue, suggestive of increasing fatty acids by lipolysis taken up in visceral fat for esterification [20.26]. These changes may constitute an important component of fat redistribution with aging.

In the current study, changes in gene expression related to lipid oxidation and energy expenditure were induced during aging. Gene expression of PDK2, which lowers glucose utilization and facilitates fatty acid oxidation [27], increased in both types of deposits with age, whereas there was no change in mCPT1 and LCAD. However, fat distribution had a significant impact on the expression of all these genes. These discrepant patterns among the genes associated with energy expenditure might be due to different mechanisms and roles played by individual genes during aging. Ravaglia et al. demonstrated that increased PDK2 might be a compensatory mechanism to increase fat mass during aging [28]. The observed difference in the mRNA expressions of mCPT1 and LCAD between the fat depots during aging is likely associated with altered energy expenditures, leading to fat deposition in visceral fat. Therefore, the net balance between the two fat deposits in lipid oxidation and energy expenditure may be altered by aging and therefore might contribute to age-related fat accretion in visceral fat. However, the mechanisms by which aging regulates lipid/glucose metabolism in different adipose tissue, fat redistribution and especially energy expenditure remains unclear. Additionally, the effects of aging on the protein levels and/or enzyme activity of the genes associated with adipose lipid metabolism were not evaluated in this study. Thus, further work is needed to elucidate these questions. To the best of the author’s knowledge, this is the first study to evaluate aging-related changes in lipid metabolism using two different fat depots in a type 2 diabetic animal model.

PPARγ agonists remodeled adipose tissue by changing the genes involved in lipogenesis and fatty acid cycling. The effects of PPARγ agonists on insulin sensitivity are mediated by fat redistribution from visceral fat to subcutaneous fat [20,26]. In this study, rosiglitazone resulted in fat redistribution and improved glucose intolerance in a type 2 diabetic animal model. Consistent with previous findings [29–31], in this study rosiglitazone also increased adipocyte cell numbers and reduced gene expression involved in lipogenesis at visceral fat deposits, thus inducing favorable conditions for fat redistribution. These changes are contrary to the aging-related changes in adipose lipid metabolism. Of note is the fact that the PPARγ agonist enhanced ATGL expression in visceral fat deposits, though it reduced plasma FFA levels. There is evidence that it also stimulates adipocyte lipolysis [22,32,33]. Because of increased fatty acid esterification, PPARγ might increase lipolysis; however, FFA release was lower magnitude than fatty acid reesterification [22,31]. It was also reported that PPARγ agonists may be associated with increased lipid oxidation and energy expenditure [18,29]. Previous studies have shown that PPARγ agonist increases glyceroneogenesis and inhibits pyruvate dehydrogenase in white adipose tissue through enhanced expression of PEPCK and PDK [21,31]. In contrast, others reported PDK2 was not affected by rosiglitazone, which was consistent with our results [34]. In the present conditions, however, the rosiglitazone had no significant effects on the PEPCK gene expression and tended to have little effect on the genes involved in lipid oxidation and energy expenditure. It is not clear why there were such differences in functional deposit specificity, but it may be linked to differences in animal models, aging, metabolic conditions, and duration of PPARγ agonist treatment or other unknown factors. Therefore, in this model, fat redistribution by the PPARγ agonist is the consequence of concerted changes in multiple pathways of adipose lipid metabolism. These data suggest that PPARγ agonists might modulate age-induced changes by remodeling adipose tissue by changing the genes involved in lipogenesis and fatty acid cycling. However, this study was limited because of the lack of data about genetic manipulation of PPARγ with aging, although this genetic manipulation may explain the direct effect of PPARγ on fat distribution during aging. The administration of PPARγ agonists might be more applicable in clinical practice. The present study extends previous findings by demonstrating the role of PPARγ agonists in adipose lipid metabolism compared with changes in age-related fat remodeling.

The mechanisms underlying age-related fat distribution are not yet fully understood. In the current study, various pathways of lipid metabolism changed with age in a rat model of type 2 diabetes. Aging stimulates lipogenesis and fatty acid cycling in visceral fat and alters lipid oxidation and energy expenditure, which leads to visceral fat deposition. Therefore, these changes might contribute to systemic metabolic dysfunction [35]. The PPARγ agonist redistributed fat mass by modifying several genes involved in age-related fat distribution. These results suggest that aging-related effects on adipose tissue distribution might be modulated by PPARγ action in a type 2 diabetic animal model.

## Supporting Information

S1 Fig **The effect of aging on the weight of subcutaneous fat/visceral fat ratio (Fig A), and OGTT (Fig B) in LETO rats (n = 4 in each group)**. There were no significant differences.
(TIF)

S2 Fig **The effects of PPARγ agonist on the body weight (Fig A), food intake (Fig B), the weight of visceral fat/subcutaneous fat ratio (Fig C), OGTT (Fig D), AUC during OGTT (Fig E) and adipocyte size distribution of subcutaneous fat (Fig F) and visceral fat (Fig G)** (*P <0.05 vs. the same depot in rosiglitazone (RGZ)-untreated OLETF rats).
(TIF)
