# Supplementary figures and images for: Depot-Specific Changes in Fat Metabolism with Aging in a Type 2 Diabetic Animal Model
Source: PLoS One. 2016 Feb 19;11(2):e0148141. doi: 10.1371/journal.pone.0148141 (PMC4760935; doi:10.1371/journal.pone.0148141)

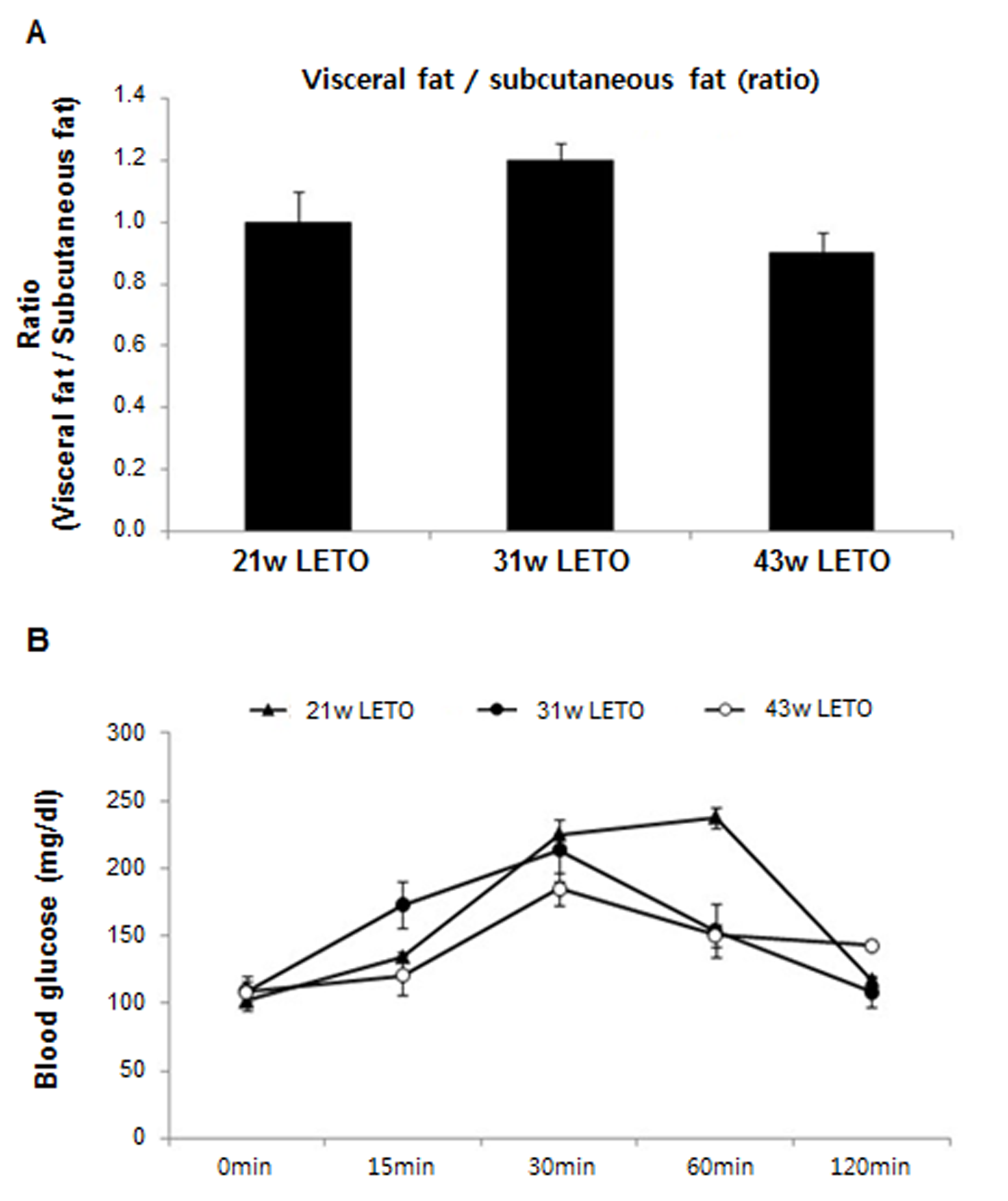

Supplement: S1 Fig — The effect of aging on the weight of subcutaneous fat/visceral fat ratio (Fig A), and OGTT (Fig B) in LETO rats (n = 4 in each group). There were no significant differences. (TIF) [file pone.0148141.s001.tif]

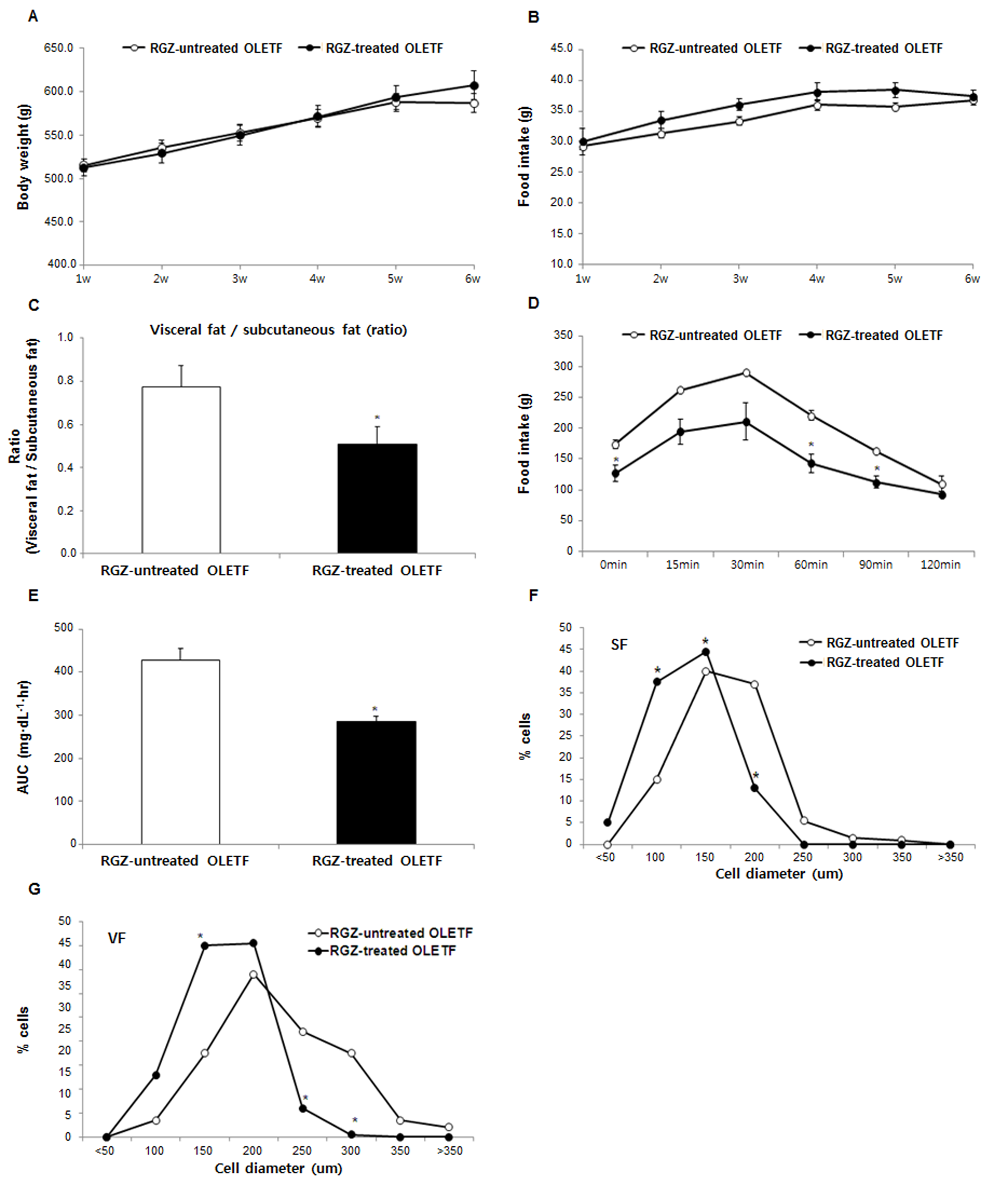

Supplement: S2 Fig — The effects of PPARγ agonist on the body weight (Fig A), food intake (Fig B), the weight of visceral fat/subcutaneous fat ratio (Fig C), OGTT (Fig D), AUC during OGTT (Fig E) and adipocyte size distribution of subcutaneous fat (Fig F) and visceral fat (Fig G) (*P <0.05 vs. the same depot in rosiglitazone (RGZ)-untreated OLETF rats). (TIF) [file pone.0148141.s002.tif]
